# Improving Pelvic Organ Prolapse Education with the iPOP Model

**DOI:** 10.1007/s00192-026-06583-x

**Published:** 2026-04-10

**Authors:** Ana Burgos, Yufan Brandon Chen, Matthew Gevelinger, William Adams, Thythy Pham, Marian Acevedo-Alvarez

**Affiliations:** 1 Obstetrics and Gynecology, Loyola University Medical Center, 2160 S 1st Ave, Maywood, IL 60153 USA; 2 Obstetrics and Gynecology, Kern Medical, 1700 Mount Vernon Ave, Bakersfield, CA 93306 USA; 3 David Geffen School of Medicine at UCLA, 10833 Le Conte Ave, Los Angeles, CA 90095 USA; 4 Urogynecology and Reconstructive Pelvic Surgery, Loyola University Medical Center, 2160 S 1st Ave, Maywood, IL 60153 USA; 5 Health Sciences Biostatistics Core, Loyola University Chicago, Chicago, IL USA; 6 Urogynecology and Reconstructive Pelvic Surgery, Endeavor Health/University of Chicago, Evanston, IL USA

**Keywords:** Education, Prolapse, Simulation

## Abstract

**Introduction and Hypothesis:**

The pelvic organ prolapse quantification system (POP-Q) can be difficult to learn, but visual aids can facilitate its learning. We aimed to evaluate the Interactive Pelvic Organ Prolapse (iPOP) model as a teaching tool for residents learning the POP-Q and hypothesized that training with the iPOP model would enhance learner confidence and proficiency in conducting POP-Q examinations.

**Methods:**

This was a pre–post intervention exploratory study. Obstetrics and Gynecology (OB/GYN) residents from one institution were surveyed regarding their understanding, comfort, and confidence teaching the POP-Q examination. They completed a POP-Q examination on iPOP models demonstrating prolapse. After an education session with iPOP models, residents again performed POP-Q examinations on the same models and completed a survey regarding their experience with the POP-Q examination. A subset of residents performed a POP-Q examination on a standardized patient. Paired *t* tests were conducted to evaluate improvements in overall POP-Q scores from the baseline assessment.

**Results:**

All eligible OB/GYN residents (*n* = 18) participated in the study. Compared with their baseline response, participants significantly improved their understanding of the POP-Q OR = 11.08, *p* < 0.001), comfort performing the POP-Q (OR = 7.10, *p* < 0.001), and confidence teaching the POP-Q (OR = 13.53, *p* < 0.001). There was overall improvement in POP-Q grid scores for the stage 1 (mean difference = + 3.00) and stage 3 (mean difference = + 2.83) models.

**Conclusions:**

The iPOP model is a useful educational tool that allows resident learners to be more confident and adept at performing POP-Q examinations.

## Introduction

The pelvic organ prolapse quantification system (POP-Q) is an objective, compartment-based system used to describe, quantify, and stage pelvic organ prolapse (POP). The POP-Q is the preferred modality for describing POP [1]. The International Continence Society (ICS), the American Urogynecologic Society (AUGS), and the Society of Gynecologic Surgeons (SGS) approved it as a standardized system for communicating details of pelvic-floor defects in individual patients. The American College of Obstetricians and Gynecologists, AUGS, SGS, and ICS all recommend performing a POP-Q examination prior to POP treatment [2]. Moreover, the Council on Resident Education in Obstetrics and Gynecology considers performing a physical examination to evaluate pelvic-floor defects an educational objective for the core curriculum of Obstetrics and Gynecology (OB/GYN) [3]. However, the three-dimensional nature of the POP-Q can be difficult to understand and thus may limit its use in clinical practice [4–6]. Therefore, visual aids and models may be helpful in its teaching by facilitating the visualization of distorted anatomy with realistic models.

**Fig. 1:**
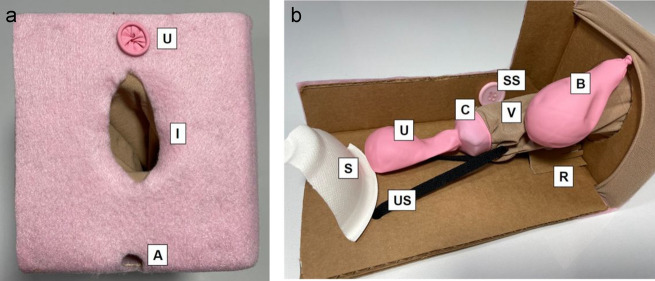
**A** Interactive pelvic organ prolapse (iPOP) model depicting external genitalia, including urethra (U), introitus (I), and anus (A). **B** iPOP model depicting pelvic structures, including bladder (B), vagina (V), cervix (C), uterus (U), uterosacral ligaments (US), sacrospinous ligament (SS), sacrum (S), and rectum (R). The iPOP model allows for the educator to mobilize the compartments to simulate different types and degrees of POP. Visit https://www.youtube.com/watch?v=J-nay3xOcCw for step-by-step instructions on how to create the model

Three models have been proposed to aid in the understanding of POP-Q, including the “Santa Claus cap” model [7], the “sock-and-tube” model [8], and the “interactive pelvic organ prolapse (iPOP) model” (Fig. 1) [9]. The iPOP model, designed by the authors, is interactive, affordable, and accessible. As the models listed, the iPOP model allows the representation of varying degrees of prolapse. However, the iPOP model has several advantages over the other models:

1. It is built to scale and can be used to practice POP-Q measurements on various types and stages of prolapse.
2. It represents the three-dimensional relationship of organs within the pelvis and those involved in POP.
3. It allows for the demonstration of surgical procedures for POP, such as sacrocolpopexy, uterosacral ligament suspension, and sacrospinous ligament fixation.

Thus, the iPOP model may serve as an excellent resource for learning POP and the POP-Q examination.

We sought to investigate the utility of the iPOP model for teaching POP-Q to OB/GYN resident physicians who had had no prior exposure to the model. The primary aim of this exploratory study was to determine if the use of the iPOP model as a teaching tool improves resident understanding, comfort performing, and self-reported confidence in teaching the POP-Q examination. The secondary aims were to:

1. Determine the content validity of the iPOP model
2. Grade the participant’s POP-Q examination grid correctness on the iPOP model and a standardized patient
3. Evaluate the usefulness of the iPOP model as an education tool

We hypothesized that learners would be more confident and adept at performing the POP-Q after learning with the iPOP model.

## Materials and Methods

**Fig. 2:**
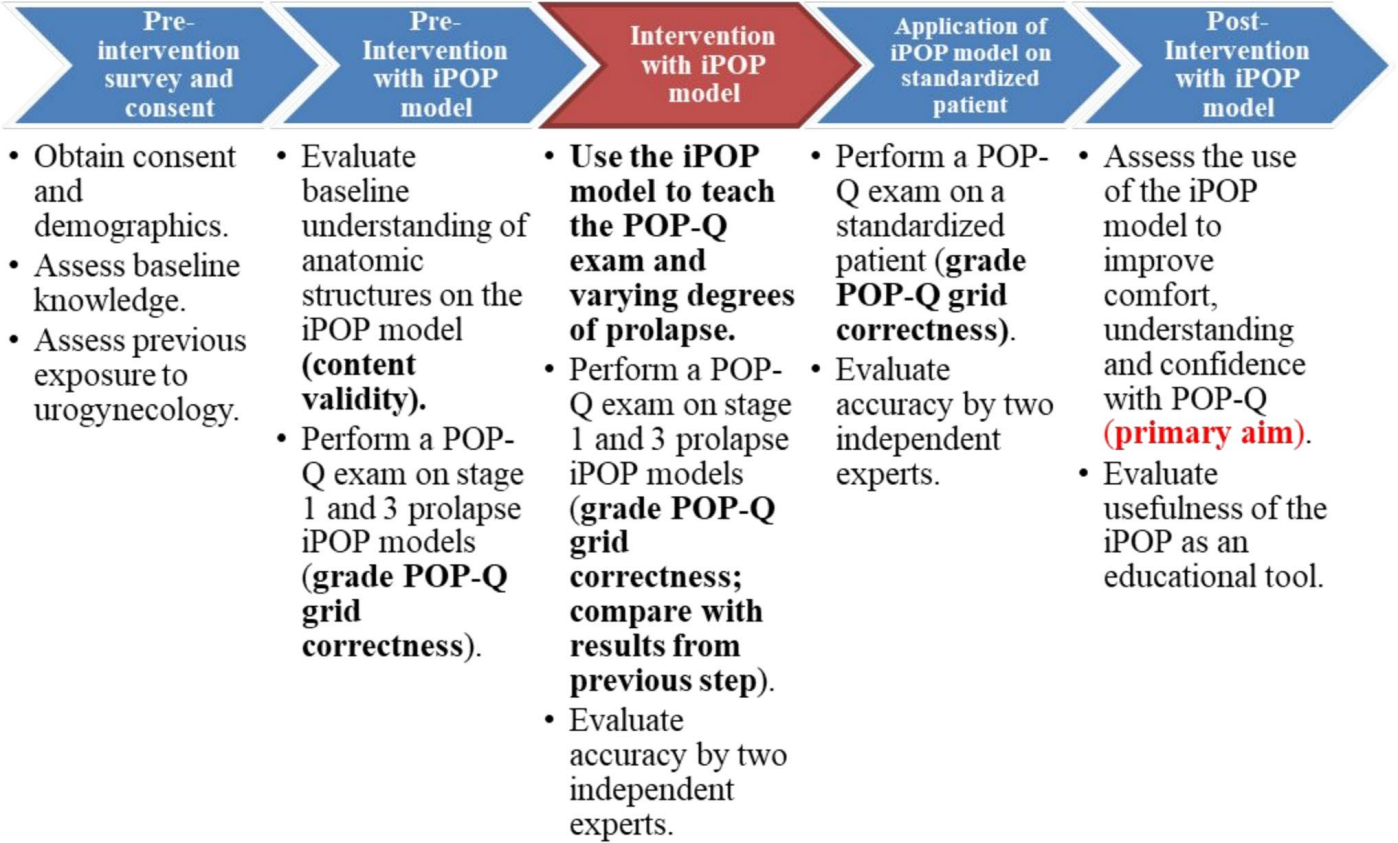
Graphical representation of the study methodology, including the goals of each step

This study was performed at a single institution between August and September 2022, and all eligible OB/GYN residents from all 4 years were invited to participate. It was deemed exempt by the Institutional Review Board. See Fig. 2 for a graphical representation of the study methodology.

### Baseline Knowledge Survey and Consent

All surveys were adapted from a previously published survey evaluating comfort and knowledge with POP-Q [8]. Residents received surveys anonymously to evaluate their baseline understanding of POP-Q, comfort performing the POP-Q examination, and confidence in teaching POP-Q. A five-point Likert scale (1 = strongly disagree to 5 = strongly agree) was utilized in the questionnaire (Appendix 1). Consent to participation in the study was included at the start of the questionnaire and assumed if the questionnaire was completed. The number of weeks on a urogynecology rotation and prior exposure to the POP-Q examination were also assessed. Residents typically rotate through the Urogynecology service during their second and third years of residency for one and two blocks of 5 weeks, respectively.

### Pre-Intervention with the Interactive Pelvic Organ Prolapse Model

After completing the pre-survey, residents were brought together during protected educational time to maximize resident participation or, if unable to attend, at a time that was convenient to each resident. Each participating resident was provided with their own iPOP model. The iPOP models were constructed from low-cost, readily available materials including fabric, foam, and balloons, assembled to scale to represent the female pelvic organs and supporting structures. The models included anatomically recognizable components representing the bladder, vagina, cervix, uterus, rectum, urethra, introitus, uterosacral ligaments, sacrospinous ligament, and sacrum. Each compartment was independently mobile, allowing controlled adjustment to simulate varying degrees and types of POP. Key anatomical structures on the iPOP models were labeled with letters (Fig. 1). Residents were asked to identify each of the labeled structures by filling out a worksheet. The content validity of the model was determined by examining the appropriate identification of anatomical structures portrayed by the model.

A separate set of iPOP models was set up to demonstrate stage 1 and stage 3 POP. To simulate specific POP-Q stages, the position of each compartment was manually adjusted relative to the hymenal ring in accordance with standard POP-Q definitions. Once the desired configuration was achieved, all components were secured in place using adhesive fixation to prevent movement during examination. Separate iPOP models were permanently configured to represent stage 1 and stage 3 prolapse and were not altered during the study period. This approach ensured that all participants evaluated identical, reproducible anatomical configurations. Residents were provided with popsicle sticks that had ruler markings (POP-Q sticks) and asked to complete a POP-Q examination on each model. Worksheets containing a POP-Q grid with all nine components of the POP-Q were provided for data collection.

### Intervention with the Interactive Pelvic Organ Prolapse Model

An education session was conducted with the use of the iPOP model as a passive and active teaching aid. Residents were again asked to complete a POP-Q grid for the same two iPOP models used initially to demonstrate stage 1 and 3 prolapse. Two experts (urogynecology-trained fellows and faculty, and co-authors of the study) independently performed a POP-Q examination on each model to evaluate the accuracy of participant-reported measurements, thus serving as a control group. Two experts were used to ensure the reliability of their measurements with the model; these measurements rarely differed between experts by ± 1.0 cm. POP-Q grids were scored based on the correctness of each cell, meaning that each correct cell on the POP-Q grid was awarded one point, for a total of nine possible points.

### Application of the Interactive Pelvic Organ Prolapse Model on a Standardized Patient

After the intervention, participants performed a POP-Q examination on a standardized patient, a trained individual providing a realistic and reproducible clinical examination scenario. Experts conducted the examination as well to establish the reference standard and assess the accuracy of participant responses.

### Post-Intervention with the Interactive Pelvic Organ Prolapse Model

After the education session and completion of the POP-Q examinations, all participants were asked to complete an anonymous survey online (Appendix 2). Survey responses before and after the intervention were compared to assess if the iPOP model served as a tool to improve resident understanding of, comfort performing, and confidence with teaching POP-Q. The post-intervention survey also included questions regarding resident perception of the usefulness of the iPOP model as a teaching tool and their likelihood of using it themselves.

### Planned Statistical Analysis

Counts and proportions were calculated for nominal and ordinal survey responses, whereas means and standard deviations were reported for quantitative survey responses. Ordinal generalized estimating equations were used to assess whether residents expressed greater agreement post-intervention regarding their understanding of the POP-Q examination, comfort performing the examination, and confidence in teaching it, compared with their baseline responses.

Paired *t* tests were conducted to evaluate improvements in overall POP-Q scores from the baseline assessment, with results stratified by post-graduate year (PGY) level. When sample sizes were limited, exact nonparametric tests were used to confirm model conclusions. Descriptive statistics were used to analyze data from POP-Q examinations performed on a standardized patient. All analyses were conducted using SAS version 9.4 (Cary, NC, USA).

## Results

### Baseline Knowledge Survey and Consent

From August to September 2022, all eligible subjects (*N* = 18) participated in the study. Two residents from the program were excluded (extended medical leave and the principal investigator on the project). There was adequate representation between classes; most participants had spent 0–5 weeks on urogynecology (Table 1). Most residents had been taught the POP-Q examination before the intervention (55.6% vs 44.4%).

**Table 1:** Survey responses (counts and proportions) from the pre-intervention anonymous survey (*N* = 18)

Survey question	Number of respondents (%)
Level of training	
PGY-1	5 (27.8)
PGY-2	5 (27.8)
PGY-3	3 (16.7)
PGY-4	5 (27.8)
Number of weeks on a urogynecology rotation	
15 weeks	5 (27.8)
10 weeks	0 (0)
5 weeks	5 (27.8)
0 weeks	8 (44.4)
I was taught the POP-Q examination before (recoded)	
No	8 (44.4)
Yes	10 (55.6)

*PGY* post-graduate year

### Resident Understanding, Comfort, and Confidence (Primary Aim)

Compared with their baseline response, participants significantly improved their understanding of POP-Q (OR = 11.08; 95% CI 3.21–38.24; *p* < 0.001), comfort performing the POP-Q examination (OR = 7.10; 95% CI 2.71–18.59; *p* < 0.001), and confidence teaching POP-Q (OR = 13.53; 95% CI 5.03–36.35; *p* < 0.001; Table 2). These results did not depend on whether participants had been previously taught POP-Q prior to the intervention (all interactions *p* > 0.05) or on the number of weeks on a Urogynecology rotation (all interactions *p* > 0.05).

**Table 2:** Survey responses (counts and proportions) from pre-intervention and post-intervention anonymous surveys (*N* = 18). Participants were asked to respond with their level of agreement with the following statements using a five-point Likert scale (completely disagree to completely agree)

Survey question	Pre-intervention (%)	Post-intervention (%)	*p*
I understand the POP-Q examination			< 0.001
Completely disagree	6 (33.3)	0 (0.0)	
Somewhat disagree	2 (11.1)	0 (0.0)	
Neutral	2 (11.1)	0 (0.0)	
Somewhat agree	5 (27.8)	10 (55.6)	
Completely agree	3 (16.7)	8 (44.4)	
I feel comfortable performing the POP-Q examination			< 0.001
Completely disagree	8 (44.4)	0 (0.0)	
Somewhat disagree	1 (5.6)	0 (0.0)	
Neutral	1 (5.6)	3 (16.7)	
Somewhat agree	6 (33.3)	9 (50.0)	
Completely agree	2 (11.1)	6 (33.3)	
I feel confident teaching the POP-Q examination			< 0.001
Completely disagree	8 (44.4)	0 (0.0)	
Somewhat disagree	3 (16.7)	1 (5.6)	
Neutral	3 (16.7)	5 (27.8)	
Somewhat agree	4 (22.2)	7 (38.9)	
Completely agree	0 (0.0)	5 (27.8)	

*POP-Q* pelvic organ prolapse quantification

There was an overall improvement in POP-Q grid scores for the stage 1 (mean difference = 3.00; 95% CI 1.78–4.22) and stage 3 (mean difference = 2.83; 95% CI 1.57–4.10) models, although the improvement depended on the PGY level of respondents (both interaction *p* < 0.05; Table 3). For the stage 1 model, the score increased by 4.30 (95% CI 2.91–5.69; *p* < 0.001) points for PGY-1 and −2 respondents (*n* = 10) but did not significantly increase for the eight PGY-3 and −4 respondents (mean difference = 1.37; 95% CI −0.35–3.10; *p* = 0.10). Conclusions were similar for the stage 3 model, where PGY-1 and −2 respondents improved by 4.50 (95% CI 3.53–5.47; *p* < 0.001) points, although the scores of PGY-3 and −4 respondents did not significantly increase over the two sessions (mean difference = 0.75; 95% CI −1.02–2.52; *p* = 0.35).

**Table 3:** Mean pelvic organ prolapse quantification (POP-Q) grid scores before and after the education session with the Interactive Pelvic Organ Prolapse (iPOP) model, stratified by post-graduate year (PGY) level. Maximum total score is 9 points (*N* = 18)

iPOP model	Mean POP-Q scores pre-intervention ± SD	Mean POP-Q scores post-intervention ± SD	*p*
Stage 1 model	1.9 ± 2.2	4.9 ± 2.0	
PGY-1 and −2	0.8 ± 1.0	5.1 ± 2.5	< 0.001
PGY-3 and −4	3.4 ± 2.4	4.8 ± 1.3	0.10
Stage 3 model	2.4 ± 2.6	5.2 ± 1.7	
PGY-1 and −2	0.7 ± 1.1	5.2 ± 1.5	< 0.001
PGY-3 and −4	4.5 ± 2.3	5.3 ± 2.0	0.35

*SD* standard deviation

**Fig. 3:**
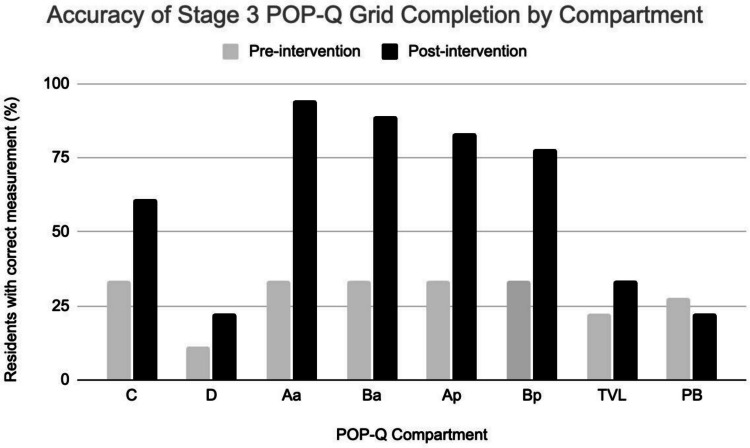
Percentage of participants who correctly measured individual compartments on the stage 3 model before and after the education session with the Interactive Pelvic Organ Prolapse (iPOP) model, stratified by pelvic organ prolapse quantification (POP-Q) grid compartments (*N* = 18)

**Fig. 4:**
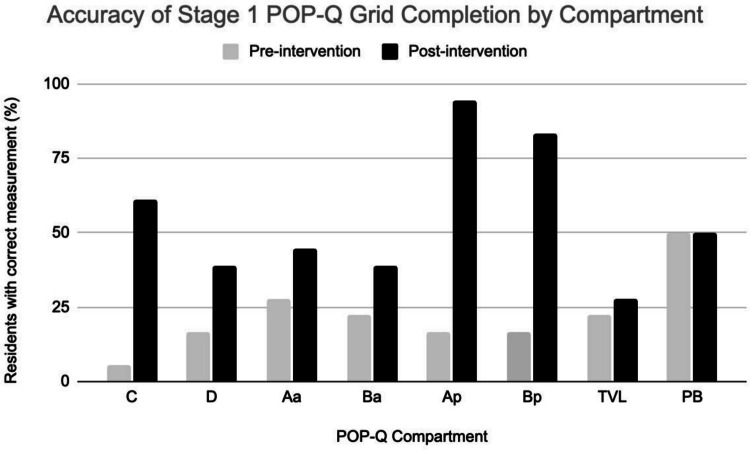
Percentage of participants who correctly measured individual compartments on the stage 1 model before and after the education session with the Interactive Pelvic Organ Prolapse (iPOP) model, stratified by pelvic organ prolapse quantification (POP-Q) grid compartments (*N* = 18)

Regarding individual compartments, 11 residents (61.1%) improved their scores in the stage 3 Aa compartment, and 10 (55.6%) improved their scores in the Ba compartment. Nine residents (50.0%) improved their scores in the stage 3 Ap compartment, and 8 (44.4%) improved their scores in the Bp compartment. Five residents (27.8%) improved their scores in the stage 3 C apical compartment and 2 (11.1%) improved their score in the D compartment (Fig. 3). Additionally, 3 residents (16.7%) improved their scores in both the Aa and Ba compartments of the stage 1 model, whereas 14 residents (77.8%) improved their scores in the stage 1 Ap compartment and 12 (66.7%) improved their scores in the stage 1 Bp compartment. Ten residents (55.6%) improved their scores in the stage 1 C compartment, and four (22.2%) residents improved their scores in the stage 1 D apical compartment (Fig. 4).

### Content Validity

Residents correctly identified all structures depicted in the iPOP model—bladder, vagina, cervix, rectum, uterosacral ligament, urethra, introitus, anus, and sacrospinous ligament—except for the sacrospinous and uterosacral ligaments, which were correctly identified by only 12 of the 18 residents (66.7%).

### Standardized Patients

Ten of the 18 participants (55.6%) performed a POP-Q examination on a standardized patient. POP-Q grids were scored in the same manner described above, with a maximum of 9 possible points. The mean POP-Q grid correctness score for all residents was 7.0 (SD = 1.5) points. Junior residents (PGY-1 and −2 residents; *n* = 6) scored on average 6.8 (SD = 1.7) points, whereas senior residents (PGY-3 and −4 residents; *n* = 4) scored 7.3 (SD = 1.3) points. When evaluating the anterior compartment, 8 of the 10 residents (80.0%) correctly answered the Aa and Ba anterior compartments; 7 residents (70.0%) correctly answered the Ap and Bp posterior compartments. Seven residents (70.0%) correctly answered the C apical compartment, whereas 9 residents (90.0%) correctly answered the D apical compartment and perineal body compartment. The genital hiatus was measured correctly by 8 residents (80.0%).

### Usefulness as an Education Tool

All residents agreed that the iPOP model was a useful educational tool, and most (*n* = 17, or 94.4%) said that they would use it to teach trainees in the future (Table 4).

**Table 4:** Survey responses (counts and proportions) from pre-intervention and post-intervention anonymous surveys (*N* = 18). Participants were asked to respond with their level of agreement with the following statements using a five-point Likert scale (completely disagree to completely agree)

Survey question	Number of respondents (%)
This model is useful as an educational tool for training overall	
Completely disagree	0 (0.0)
Somewhat disagree	0 (0.0)
Neutral	0 (0.0)
Somewhat agree	5 (27.8)
Completely agree	13 (72.2)
I am likely to use this model to teach other residents/learners	
Completely disagree	0 (0.0)
Somewhat disagree	0 (0.0)
Neutral	1 (5.6)
Somewhat agree	9 (50.0)
Completely agree	8 (44.4)

## Discussion

The POP-Q system is routinely used in research to describe the degree of POP; however, not as commonly in usual clinical practice [4]. Given its three-dimensional nature and less than intuitive structure, it may be difficult to learn. Visual aids and models can be helpful to better understand POP and the POP-Q examination. There have been low-fidelity models that have been described previously for teaching the POP-Q examination [7, 8]. In this study, we evaluate the use of the iPOP model as a realistic model of the female pelvis for learning POP and POP-Q.

When surveyed before and after an education session on POP and POP-Q utilizing the iPOP model, OB/GYN residents reported improved understanding of the POP-Q examination, increased comfort performing the POP-Q examination, and enhanced confidence teaching the POP-Q examination. This held true even with residents who had previously been taught the POP-Q examination or had completed a Urogynecology service rotation. Moreover, residents perceived the iPOP model as a useful educational tool that they would like to use with new learners. This was consistent with our proposed hypothesis.

When evaluating the correctness of the POP-Q grids, there was an overall statistically significant improvement in scores amongst all residents at both stages of prolapse that were studied. However, when stratified by junior versus senior level of learners, junior learners had a statistically significant improvement, whereas senior learners did not. This indicates that junior learners may find greater benefit from the iPOP model for learning POP and POP-Q. Senior learners were more likely to have had exposure to the POP-Q examination and already have a solid baseline understanding of how to perform it, which likely contributed to not having a significant improvement in their scores. However, despite not having improvement in their POP-Q grid correctness scores, senior residents subjectively felt more comfortable performing the POP-Q examination, which is what will ultimately impact their patient interactions.

Several physical and simulation models have been developed to support teaching of the POP-Q examination beyond traditional didactics. One early example is the “sock-and-tube” model, which uses simple materials such as socks and cardboard tubing to construct a three-dimensional POP-Q model for hands-on resident training; residents in the original evaluation reported increased understanding, comfort performing, and confidence teaching the POP-Q examination after using this model [8]. The “Santa Claus cap” model similarly uses an inverted cap mounted on a frame to represent the vagina and prolapse, allowing learners to practice spatial interpretation of POP-Q points [7]. More recently, dynamic, high-fidelity, three-dimensional pelvic models have been described that significantly enhance the comfort, objective POP-Q performance, and interest in urogynecology of learners [10]. In contrast to these existing tools, the iPOP model combines anatomical realism, controlled prolapse staging, and reproducibility at a low cost, offering a practical middle ground between simple physical teaching tools and more complex simulators for incorporation into resident curricula.

Similar to the “sock-and-tube” model studied by Parnell et al. [8], the iPOP model improved residents’ understanding, comfort with performing, and confidence in teaching the POP-Q examination. Compared with residents in the study by Parnell et al. using the “sock-and-tube” model, however, our residents reported greater gains in these areas after learning with the iPOP model. Additionally, POP-Q grid accuracy improved following training with the iPOP model. Overall, residents had a positive experience using the model as an educational tool.

When tasked to identify the anatomical structures of the female pelvis represented in the iPOP model, residents correctly identified all structures except for the sacrospinous and uterosacral ligaments. Senior residents were more likely to correctly identify these structures, which was likely due to greater exposure to urogynecological procedures. This suggests that the iPOP model might have content validity, but larger studies are necessary to further investigate this. The model provides a realistic, recognizable, and dynamic representation of most female pelvic anatomical structures.

Importantly, learners were able to apply skills acquired through training with the iPOP model to POP-Q examinations performed on standardized patients, suggesting effective transfer of knowledge to a clinical examination setting. Although long-term retention was not directly assessed, hands-on interaction with a three-dimensional, anatomically representative model may promote deeper conceptual learning and skill durability. Future studies incorporating interval reassessment or repeated exposure would be valuable to evaluate knowledge retention over time.

Strengths of the study include evaluation of both objective and subjective measures regarding proficiency with the POP-Q examination. Junior resident subjects demonstrated objective improvement in their correctness of POP-Q grids and also reported improved subjective understanding of the POP-Q examination, comfort with the POP-Q examination, and confidence in teaching the POP-Q examination. Additionally, the real-world application of these skills was tested by performing the POP-Q examination on standardized patients. All eligible OB/GYN residents in the program were included in the study. Moreover, two experts were used to evaluate the content validity and the correctness of the POP-Q grids, strengthening the validity of this model.

This investigation was performed within a single residency program, increasing the risk of type II error and limiting generalizability to other training settings. Participation in standardized patient examinations was limited by clinical scheduling demands. Interest in urogynecology as a career was not assessed and may influence baseline proficiency with POP-Q examination. As an exploratory educational study, these findings should be interpreted as hypothesis generating, and future studies with larger, multi-institutional cohorts are needed to further evaluate the educational effectiveness of the iPOP model. Another limitation is not including a comparison between the traditional teaching of POP-Q using two-dimensional drawings and the hands-on educational session with the iPOP model, or having a minimal clinically significant comparison published in prior studies.

Future directions could target medical students, as the effect size is likely to be higher than with resident subjects. Performing an interval resident assessment would also validate the ability of the model to promote retention of POP-Q knowledge. A comparison between learning with the iPOP model and other traditional methods such as two-dimensional graphics, or comparing with the “Santa Claus’ cap” model [7] or the “sock-and-tube” model [8] could be performed in the future as well. The iPOP model could also be used for patient education.

The POP-Q provides a standardized measuring system, allowing objective communication among providers clinically and through research. Although it is used routinely in the literature, some urogynecology providers report not using the POP-Q routinely in clinical practice, in part because of the difficulty in conceptualizing the three-dimensional nature of prolapse and POP-Q. The iPOP model is a dynamic, realistic three-dimensional model of the female pelvis that can be used to teach resident learners the POP-Q examination and enhance their understanding and performance of the examination. If the process of learning the POP-Q is enhanced with easy-to-use realistic models, more providers may learn it. This would increase the use of the POP-Q to describe POP not only amongst urogynecologists but also amongst generalist OB/GYN providers, non-OB/GYN providers, and even patients.

## Data Availability

The data that support the findings of this study are available from the corresponding author upon reasonable request.

## Appendix 1: Pre-Intervention Survey Questions

The objective of this study is to evaluate if the iPOP model is a helpful educational tool that serves to improve resident understanding, comfort performing, and confidence in teaching the POP-Q examination. Participation in this study involves answering the following survey questions and participating in an education session with the iPOP model. Participation is voluntary and may be discontinued at any time.

- Level of training (residency year).
- Number of weeks on prior urogynecology rotations
- I was taught the POP-Q exam before.
- I have had experience performing the POP-Q exam.
- I understand the POP-Q exam. I feel comfortable performing the POP-Q exam.
- I feel confident teaching the POP-Q exam.

## Appendix 2: Post-Intervention Survey questions

- I understand the POP-Q exam.
- I feel comfortable performing the POP-Q exam.
- I feel confident teaching the POP-Q exam.
- I will use POP-Q more in my general practice.
- After today’s session with the iPOP model, I understand the POP-Q exam better.
- Practicing on this model could help improve my performance of POP-Q exams on patients.
- This model is useful as an educational tool for training overall.
- This model is useful to improve existing skills for the evaluation of pelvic organ prolapse.
- This model is useful to obtain new skills for the evaluation of pelvic organ prolapse.
- I am likely to use this model to teach other residents/learners.
